# Exploring the Experience of Living with Pain after Spinal Cord Injury: A Qualitative Study

**DOI:** 10.1155/2024/9081530

**Published:** 2024-02-02

**Authors:** Mokgadi Kholofelo Mashola, Elzette Korkie, Diphale Joyce Mothabeng

**Affiliations:** ^1^Department of Physiotherapy, School of Therapeutic Sciences, Faculty of Health Sciences, University of the Witwatersrand, Johannesburg, South Africa; ^2^Department of Physiotherapy, School of Healthcare Sciences, Faculty of Health Sciences, University of Pretoria, Pretoria, South Africa

## Abstract

A spinal cord injury is a life-changing experience that results in functional limitations and an increased risk of secondary health conditions. People with spinal cord injury identify pain as the most devastating health problem following their injury that not only affects their social life but their mental well-being as well. This study is aimed at exploring the lived experience of living with pain by community-dwelling manual wheelchair users with spinal cord injuries. An explorative qualitative design was used to explore their experiences. In-depth interviews were recorded and transcribed, and the data were analysed using inductive thematic content analysis in the MAXQDA v2020. Fifteen manual wheelchair users with paraplegia participated in this study, and four themes were identified from their experience of living with pain: pain constantly lurks, pain is worse than the direct consequences of the SCI, pain is restrictive, and life continues despite the pain. Categories and subcategories included the participants being one with the pain; pain interfering with sleep; feelings of anger, isolation, and suicidal ideation; and uncertainties about what the future holds living with pain. Living with pain after SCI is a challenging feat, and effective management of pain is necessary to improve not only functioning and mobility but also mental health and life satisfaction.

## 1. Introduction

A spinal cord injury (SCI) brings tremendous change to the life of the affected individual that can vary depending on the type and severity of the injury [1, 2]. Expected functional outcomes following a SCI is dependent on the level and completeness of the injury. A SCI could result in partial or full sensory and/or motor loss below the level of injury that involves the upper limbs, trunk, and lower limbs (tetraplegia) or partial trunk and lower limbs (paraplegia). The preservation of sacral function determines whether the injury is complete or incomplete [3]. People with SCI (PWSCI) struggle with secondary health conditions (SHCs) such as pressure ulcers, urinary tract infections, and respiratory complications which lead to readmission back to the hospital and sometimes death [4, 5]. Pain is one of the most problematic SHCs, and persistent, severe pain is common after SCI [6, 7].

Pain has been reported in 11 to 94% of PWSCI, often commencing within the initial six months after SCI with the possibility of being aggravated over time [8, 9]. Pain has a notable impact on the lives of PWSCI beyond the physical consequences of the SCI, and pain is known to reduce health satisfaction and quality of life (QOL) [10]. It is common for pain to be reported together with other SHCs like pressure ulcers, muscle spasms, or depression [11]. For example, the coexistence of pain and depression is associated with higher severity and persistent conditions over time, as well as the increased use of specialised SCI care [12]. Some PWSCI may magnify their pain, which is associated with pain severity, mood, and pain interference [13]. This, in turn, may further increase the pain, increasing the negative mood and anxiety [14] and resulting in pain-avoidance behaviour [15].

A South African survey reported an 18.3% prevalence of chronic pain in able-bodied adults [16], with up to 85% in PWSCI [17]. Challenges that PWSCI face and their experience of overall life after SCI are well-researched in high-income countries [2, 18, 19], and research is recently being made available in middle-income countries such as Brazil, [20], Ghana [1], and South Africa [11, 21]. Pain is a personal experience, and due to the diverse cultures in Africa, it would be remiss to presume that the lived experiences of pain by PWSCI across the globe are similar. Able-bodied African Americans are found to report more pain and suffering when compared to their white counterparts [22, 23]. However, there is limited information on the African population, especially those with SCI, as the culture, gender, and African spirituality influence the meaning, perception, and expression of pain [24]. This study is therefore aimed at exploring the experiences of living with pain after SCI in Gauteng, South Africa.

## 2. Materials and Methods

### 2.1. Study Design and Author Reflexivity

This study forms part of a phenomenological qualitative approach of a mixed-method study that aimed to develop a guided pain self-management intervention framework for pain [25]. The interpretative phenomenological analysis (IPA) approach was used to explore the lived experience of pain after SCI as supported by Alase [26] and Tuffour [27]. The first author, who was the primary investigator, identifies with the relativist stance of ontology, with the belief that our reality is relative according to how we each experience it. We as individuals may behave differently in one setting when compared to another, and therefore, the patients we treat in-hospital are only a fraction of the person they are, as compared to when they are in their comfort zone in their homes. Behaviour, attitudes, and beliefs are fundamental to what makes us unique individuals. As a subjectivist where epistemology is concerned, the first author believes that individuals interpret their meaningfulness of life in a way that makes sense to them in an individualistic form. A SCI may alter an individual's outlook on life, mindset, and plans, but the core of what makes them “them” may not change. The first author kept a reflexive journal as a form of bracketing, to limit the influence of personal beliefs on the findings reported [28].

### 2.2. Study Participants

Only manual wheelchair users with paraplegia were included in the mixed-method study, and this paper reports findings from the explorative qualitative design that was used to explore their lived experience with pain. People with tetraplegia were excluded as the authors aimed to also determine the factors associated with shoulder pain in fully innervated upper extremities [25]. Participants were purposively sampled from our preceding quantitative study that investigated the presence of pain in manual wheelchair users discharged from one private and four public rehabilitation hospitals in Gauteng, South Africa [25]. The quantitative study included 122 participants who resided within 500 km of their discharging hospitals [17]. 85% of these participants reported the presence of pain, and participants residing within 150 km of their discharging hospitals were purposively sampled to allow the first author to visit them again for the qualitative data collection. Thirty-nine participants were recruited to be interviewed and 21 consented to the interview. The first author is fluent in English, Sepedi, and isiZulu, three of the 11 official languages of South Africa, and the interviews were conducted in either of these three languages.

### 2.3. Procedure

Consenting participants were visited at their homes, and semistructured audio-recorded interviews were conducted by the first author. Demographic data were captured after the interview, where an interview guide with open-ended and probing questions was used to ask the participants how they experienced life with pain and how they think the pain will affect their future if at all. Participants were asked to speak about their experience of life living with pain after SCI, with probing questions including the location of pain, how it feels at best and worst, as well as how the pain often presents itself. Participants were also asked if they think the pain will affect their future, if at all. A pilot study was conducted with two participants to test if the questions were understandable and if there were changes that needed to be implemented to improve the process of data collection or the clarity of the questions. No changes were necessary, and findings from the pilot study are included in the main findings. Fifteen interviews, which lasted approximately 30 minutes, were conducted from October 2019 to February 2020 until no new views, opinions, categories, or codes emerged. The investigator observed through the course of interviewing that the same ideas, views, and themes came out repeatedly by the eleventh participant, and four more participants were interviewed before the first author determined that data saturation was reached after no new information emerged [29, 30].

### 2.4. Data Analysis

All the recorded interviews were translated and transcribed verbatim by an independent company as suggested by Tindall et al. [31], and the first author checked all transcripts and compared them to the audiotaped recordings to verify their accuracy. To ensure accuracy and trustworthiness, the non-English transcripts were also translated back to their original language and the first author checked that the translations did not lose the meaning of the statements. Inductive thematic analyses were performed to code the data. The first author and two external academics, who held PhDs and published qualitative studies in the field of pain management and SCI, each coded the first two transcripts separately to ensure intercoder reliability before the rest of the transcripts could be coded [32]. There was no predetermined quantification of agreement between the first author and the two external academics, as the selected approach aimed to emphasize the need to achieve consistency between the three individual's coding and not the extent of the agreement. This choice is supported by Cofie et al. [33], who further explained that such a choice encourages not only authenticity during the data analysis process but also reflexivity. A discussion was held between the first author and the two external academics, and similar codes were grouped to form subcategories, categories, and themes, and a coding framework was agreed upon thereafter. The coding process was assisted by the MAXQDA software version 2020.

### 2.5. Ethical Considerations

This study was registered with the South African National Health Research Database (reference number GP201806005) and received ethical approval from the Faculty of Health Sciences Research Ethics Committee, University of Pretoria (approval number 125/2018). Written informed consent was obtained from all the participants in our study, and pseudonyms are used in place of participants' names to ensure confidentiality. In one instance, the interview was paused when the participant became distressed before opting to continue and the participant was subsequently referred to the psychology department of their discharging hospital.

## 3. Findings

Fifteen participants were interviewed, and their mean age was 43.8 years (SD 12.05), and the mean age when injured was 34 years (SD 11.92). The mean duration of living with SCI was 9.73 years (SD 9.18). The full demographic picture is depicted in Table 1. Participants reported up to four painful areas, and the most severe pain that was expressed is included in Table 1, and the mean pain severity was 6.7/10 (SD 1.80).

**Figure 1:**
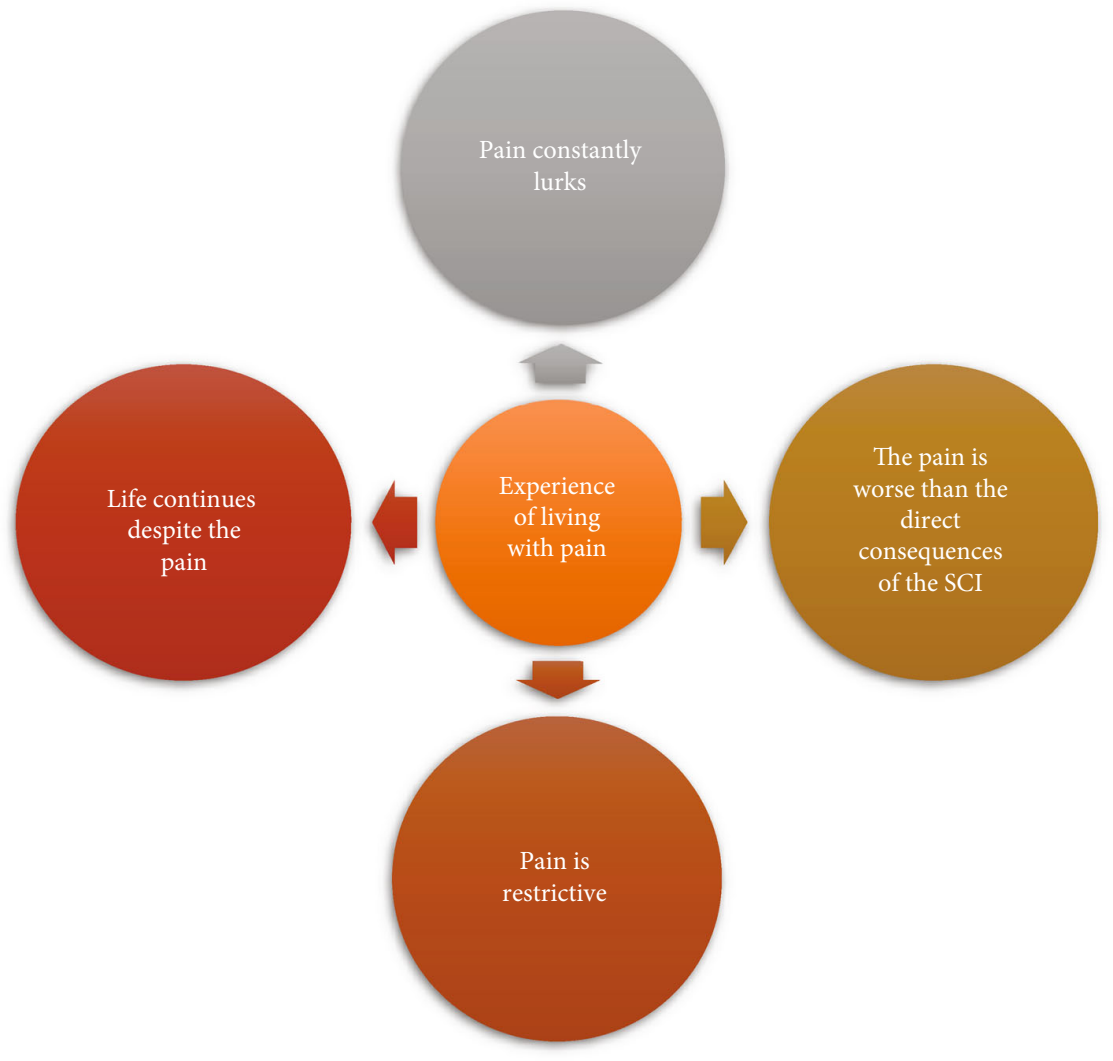
The themes identified in this study.

Four themes were identified from the findings (Figure 1), and their categories, subcategories, and quotes are presented in narrative form. The quotes are identified by the participants' pseudonyms, age and gender, and duration of living with SCI in the first theme.

### 3.1. Theme One: Pain Constantly Lurks

Most of the participants expressed that they have been living with their pain for many years since they had the SCI, suggesting that pain is a common secondary health condition immediately following SCI. With SCI being a life-long injury, this finding implies that pain and SCI are similar to two acquaintances walking hand in hand throughout the journey of life:

*I've had it since the beginning … it's always there*. (Angela, nine years living with SCI)

Not only is the pain insistently present, but some participants also described it as lurking in the background as if in wait to either pounce or let the person know that it is there to stay:

*The background pain, it has been there. It's part of me*. (Andries, 13 years living with SCI)

Most of the participants expressed that the pain fluctuates in intensity not only throughout each day but also during individual days, suggesting that although the intensity of the pain may differ, the participants could always count on the pain to be present daily:

*Since I woke up in the hospital it's been the same feeling, varying fluctuations of it, but the same thing constantly*. (Anthony, four years living with SCI)

Most of the participants said that the pain did not subside with increasing years since their injury. This finding adds to the complexity of pain as not only is it continually present from the beginning of the injury, it is unpredictable at times in its presence and even worsens in severity with more years of living with SCI. For example, Alice, who was living with SCI for four years, simply said “It's getting worse.” When asked if the pain was getting better.

### 3.2. Theme Two: Pain Is Worse than the Direct Consequences of the SCI

Pain affected so many aspects of life, and both categories and subcategories were identified to derive this theme. For most participants, their lives were not at a satisfactory level not because they were living with SCI but because of the unbearable pain they had to live with:

*It's been a huge issue for me. It's probably the worst thing I have to deal with, after this spinal cord injury. I personally feel like the pain is worse than not walking*. (Anthony)

To further understand how pain affects the lives of the participants, the quotes gave rise to the following categories that were affected by pain: physical, emotional, mental, and relationships.

#### 3.2.1. Physical Health

In some instances, participants opted not to propel their wheelchairs over rough and uneven terrain so that they could avoid aggravating their shoulder pain. This then restricts them to only using their manual wheelchairs on even surfaces, which may not always be practical:

*On sports days, I would go from the car park to where the field starts and I'll sit there in the blazing heat, the rain, whatever the case is because I know if I go to the other side of the field I'm going to cause myself pain*. (Andrew)

There are multiple SHCs that may develop and coexist following SCI, and some participants reported how pain not only coexisted with spasms but aggravated the frequency and severity of the spasms:

*Then I stop and just be still … once I try to move, the spasm also are against me*. (Andries)

#### 3.2.2. Emotional Health

The presence of pain may dictate how the participants could function in their daily life (for example propelling a wheelchair over rough terrain), or even affect the speed at which the activities could be executed (due to stopping to accommodate the pain), to a point that some participants became impatient with themselves:

*Since I started having this pain, I feel like it's very hard because I get impatient with myself …*. (Abraham)

Frustration in oneself does not fall far from frustration in others, and unsurprisingly, some participants expressed how they often lost patience with friends and family because of their experience of pain. This leads to some feeling guilty about how they treat those around them:

*It's not nice to live in pain. When you're in pain you become moody and lose patience with people. So, it's not nice*. (Abigail)

Andrew suggested that the frustration would escalate to the point where he would begin being angry towards others, not because of their possible actions but because he was in pain.

*I'm going to become angry towards people because now I'm in pain*. (Andrew)

It was challenging for the participants to try to cope with the pain, as well as the emotional turmoil that the pain caused, making some patients emotionally distressed because they were experiencing many emotions simultaneously:

*It's been difficult because your moods are always up and down, up and down. You're always irritable because of the pain, you feel like crying*. (Alice)

Alice further described how she wished that she could “let it all out” somehow if only to have any pain relief:

*I don't know, sometimes you feel like screaming, just maybe if I scream the pain will vanish with my voice …*. (Alice)

The effects of pain on emotional health were so significant in some participants, that they mentioned how they could not function because of the resultant “weight on their shoulders.”

*When I feel the pain and my mood is low, then I can't function because it's the pain, and the stress, and all these things together make me malfunctional [sic].* (Alice)

#### 3.2.3. Mental Health

Poor emotional health may often go hand in hand with poor mental health, and our participants expressed various ways in which the pain negatively affected mental health. Lack of concentration was expressed by some participants, in a rather nonchalant way:

*It even disturbs my concentration because anyway when you have pain you cannot concentrate, right?* (Abigail)

Most of the participants' pain forced them to lose focus on activities they needed to do, or pay attention to, and purely focus on the pain at hand, leading to a perceived loss of friends in the process:

*It affects my life a lot because when coming to pain, let's say maybe I have a friend, I'm talking to that friend and then the pain comes along, my mind will focus on that pain so, my attention like, it's gone on the particular subject. So, in that way, I'm losing friends because I can't focus, I can't pay attention, I can't listen to them ….* (Archie)

Findings of social relationships were intertwined with mental health in such a way that relationships with friends and family were not devoid of expressions of mental health. For example, some participants like Aaron felt obligated to isolate themselves so that they could attend to the pain. In Archie's case, isolating himself resulted in feelings of loneliness because he was the one “feeling the pain” and therefore felt obligated to go through this experience of pain alone:

*When it attacks me, I feel like I should be alone. Like, close the door and sit by myself and stretch for some few minutes by myself.* (Aaron)

*Look at me now, I'm alone, this is how I deal with pain … So, I deal with pain by spending most of my time alone.* (Archie)

Going through a painful experience alone, even when it is pain itself, seems to be a heavy burden to bear. Some participants felt that they had to put up a façade and not let friends or family see the hurt that lies beneath the outward appearance:

*It must always appear that dad is recovering, not getting worse.* (Alex)

*You always have to come up to be strong and you don't want to show everyone that you're actually weak, but then inside you're actually dying.* (Alice)

In cases where participants attempted to drop the façade and show the world what they were going through, the response was not positive from both friends and family, causing outbursts and self-doubt in some participants:

*A lot of my friends still don't quite grasp the concept of permanent pain to a point where literally, my best friend that was my best man at my wedding, got upset with me one day because I couldn't see him because I had pain. He said, you always got pain and I kind of lost my cool then, but I do always have pain.* (Anthony)

*My late husband used to tell me that I was crazy. I thought that I'm not going to come back to work, I'm not going to have kids again, I'm not going to have this life ….* (Amanda)

Some participants expressed how their emotional health caused by the pain resulted in so much worry, that they developed symptoms of anxiety and headaches:

*[The pain] affects me because you find that I cry all the time, like I don't know what to do with myself. On the other hand, it disturbs me, it causes headaches because I'm always worried about when will these pains get better.* (Abigail)

In some cases, the worry of not being able to execute activities because of pain became so cumbersome that the only way not to worry was to simply give up:

*I just gave up because I cannot do certain things. If I could do a lot of things, I wouldn't worry too much.* (Adam)

When one has pain constantly on their mind, it comes as no surprise that the individual would overthink their experience of pain. This overthinking has involved brief thoughts about death to no longer feel the pain:

*Another thing is thinking a lot … Thinking, it's a lot. You end up thinking why do I feel so much pain, why don't I just die …? So, but I also think I need help so I can be well … because I only live once.* (Abraham)

Suicidal ideation however was evident in our findings, with some participants feeling as if the only way to stop the pain was to stop living:

*Sometimes [the pain] makes me feel like I want to stop, like I want to always be sleeping and not wake up, so I don't feel the pain.* (Abigail)

### 3.3. Theme Three: Pain Is Restrictive

Although pain, in general, affected the lives of the participants, it was severe pain that interfered with aspects of their lives, namely, activities of daily living, sports, socialising, relationships, sleep, and work.

#### 3.3.1. Activities of Daily Living

Simple day-to-day tasks were challenging to complete for some participants due to the increased severity of the pain:

*When it gets severe, I cannot do anything … I just leave what I'm doing at the time and hold this leg because it gets too much ….* (Abraham)

After their SCI, participants continued to partake in household chores; however, severe pain dictated the actions of most of the participants and warranted that they stop the chores to ease the pain.

*Like most of the time I sometimes help my sister, we'd be tending the garden outside, when I have the pain I cannot. To sweep, to clean up the yard, I can't because when I have that pain, I have to relax, leave what I am doing and sit still.* (Aaron)

The presence of severe pain even hindered some participants from bathing as they would normally, affecting their esteem:

*If like maybe I have to go to work but I feel a lot of pain, I can't take a full bath… so I will just make sure that I bath where people see and then I go to work … It's bad ….* (Archie)

One participant expressed how changing the way of doing things because of the pain resulted in him feeling more limited than the SCI restrictions, underpinning the seriousness of severe pain:

*So, the pain limits you, like your transfers and forces you to try and find alternatives ways to not injure yourself … You're going around avoiding those things, so you end up with more limitations than you theoretically already have.* (Andrew)

#### 3.3.2. Sports Activities

Participating in leisure activities may improve our general mood and even reduce it; however, this was not possible for Andrew who enjoyed playing wheelchair sports but could not return due to the severe pain with movement.

*As we're sitting right now, there is a bit of pain … I can't play wheelchair rugby now because I'd be like, no sorry.* (Andrew)

#### 3.3.3. Socialising and Relationships

Socialising with others was too difficult a feat when the pain was too severe to bear, even for events the participants would not have missed had they not been in any pain:

*There's quite a few times where I've missed certain activities, going out for birthdays and stuff like that because I was just in too much pain to go down the stairs again and getting to the car and drive ….* (Albert)

Adrian expressed how he used to go out often to socialise with friends but the severe pain has interrupted this and further interferes with his intimate relationship, which he expressed with long pauses in his responses as if his mind had taken him far away when responding:

*It's affected my gallivanting quite a bit over the last year…I used to spend some time with my girlfriend … We used to travel … spend the weekend … I can't do that anymore … very often I say, hey baby, I'm sorry I'm just not going to do anything, I'm going to climb into bed for an hour or two, which is not nice with a girlfriend, you know.* (Adrian)

#### 3.3.4. Work

Severe pain interrupted not only the opportunities for employment but also performing allocated duties at work. Such disturbances may need an understanding line manager when working days and deadlines are missed:

*Look, basically when [the pain] gets very bad, I don't go to work. It has affected my work as well and I've taken off more time this last year than I've taken off in the last 39* years. (Adrian)

*Sometimes you find I'd be working and feel like just switching off the computer and sit for a while. Then when I feel better, I start working.* (Abigail)

#### 3.3.5. Sleep

When pain is severe, it may interfere with sleep by waking one up when asleep, or being unable to sleep because of the pain, which may make the experience of pain even more unbearable:

*… You cannot just sleep because [the pain] can attack ….* (Abel)

*Sometimes in the morning I'm woken up by the pain, this pain forces me out of sleep.* (Amanda)

### 3.4. Theme Four: Life Continues despite the Pain

There were varying opinions about what the future of living with pain may hold for the participants, and their responses varied from being determined to have a purposeful life despite the pain, to being worried, uncertain, and optimistic about the future.

#### 3.4.1. Uncertain

Some participants did not know how their pain could affect them in the future if at all, and perhaps living in the moment was all that was needed in an uncertain future:

*I've never thought about that, you know. I just know I've got pain, but I haven't thought maybe it can become worse … I haven't come to that decision.* (Amanda)

#### 3.4.2. Determined

The future is unknown, and some participants were more than willing not to let the pain control their lives:

*I don't think I'll give it that chance [to affect my future]. I don't know what way it would have come but I'm not prepared to entertain it.* (Angela)

Living with pain is not easy; however, some participants found a way to live with pain, even without the pain relief, displaying acceptance of life with pain:

*Yes, pain is pain, it's painful, but when you have it and it won't be reduced when you want it to. Just let it be because no matter how much digging, if it doesn't disappear at that time, I think it doesn't make a difference. It's better to just let it be and it'll just be reduced by itself.* (Alex)

*I can continue with my life for the most part. I can stop drinking or come home early from social events or things like that because of extreme pain, but I still continue with my life.* (Anthony)

The future seemed less daunting for participants who had a semblance of control over the pain. For example, pain management techniques that were effective in reducing the pain gave some participants an idea that the future may not be so bad despite the presence of pain:

*I've learned now to control it psychologically, I put it at the back of my head.* (Amanda)

*Yeah, I don't think there will be a problem as time goes on as long as I take the medication the way I have been told, I don't think there'll be a problem.* (Abel)

Pain is seen as the better devil when compared to other SHCs that participants have healed from, making the future with pain better than pressure ulcers:

*On the one hand, the pain is very debilitating and annoying and constantly in the back of my brain, on the other hand, it's a good thing because what's worse? Living with the pain or having a fat pressure sore and having to spend weeks and months in bed maybe.* (Adrian)

Some participants not only accepted life with pain but that there is no way around avoiding old age with a pain-riddled body. Be that as it may, some participants were resolute in that life is for living:

*As you get older obviously the wear and tear we're putting on our shoulders at the moment, as we get older our joints are going to get weaker, my arms are going to get weaker which is going to increase the pain and the damage done to our shoulders. And more damage, more pain and it's a vicious cycle, but it is inevitable.* (Andrew)

#### 3.4.3. Worried

Not all participants were as resolute and worry-free about their futures, and Alice in particular, was worried about being intimate and being able to have children with the pain she was experiencing:

*I'm afraid of, well, I'm dating. Sometimes I feel like my sleeping with my boyfriend it might be affected because of the pain. Like, I won't enjoy things like I did before … I still want to have babies, so I'm not sure if I'll be able to with this spinal cord pain.* (Alice)

Living with both pain and SCI is challenging, and both the lack and presence of family support worried some participants:

*The doctors tell them [the family members] what to do and they agree but when they get home, they don't do it. That's what worries me.* (Adam)

*The problem is that a lot of the time the person that I do a lot of things with is my husband, and he is also getting old, he's over 60 … I know his backache was caused by me and I try to protect him.* (Angela)

Not having the pain relieved now makes some participants worry if the pain will ever really alleviate, as this may affect their independence, especially if the pain worsens:

*I'm always worried about when will these pains get better.* (Abigail)

*I still want to be on my own but I'm not sure if I'll be able to with the pain.* (Alice)

Lastly, participants who were hoping to be employed in the future worried about what working with pain may entail, as the current treatment methods did not seem feasible in a work setting:

*… Let's say now I found a job, and the pain starts, I must leave the work I'm doing and then concentrate on it. That is what is going to be my problem. Let's say there's no bed, now I have to try and get off the wheelchair and get on the floor, do the stretches and you find that I'm reducing the time I could spend working.* (Aaron)

#### 3.4.4. Optimistic

Some of the participants were hopeful about the future with pain, as experiencing pain meant possible sensory return:

*Getting the pain, I think was to my advantage to see that maybe something is happening. Like, I'm getting the sensation back.* (Abel)

## 4. Discussion

Participants in this study predominantly complained of neuropathic pain, which was characterised by burning or pins and needles behaviour, similar to the literature [9, 34]. Most of the participants reported high pain severity consistent with findings by Rodrigues et al. [14] and were representative of the 6.7/10 mean severity in the population where these participants were purposively sampled from [17]. This study explored the lived experience of life living with pain by manual wheelchair users with paraplegia and found four themes that were interlinked, namely, pain constantly lurks, pain is worse than the direct consequences of the SCI, pain is restrictive, and life continues despite the pain. The experience of pain is a personal journey, and findings from our study confirm that pain is a persistent phenomenon in the spinal cord population [9]. The background pain found in this study is concurrent with findings by Hearn et al. [35], whose participants described pain as “the devil in the corner.” Furthermore, similar to the literature, the severity of pain does not diminish with increasing years of living with SCI, instead, it increases [36–38]. The survival rate of PWSCI has improved in recent times, increasing the life expectancy and consequently giving rise to living with SHCs for longer [1, 39]. There is, therefore, a need to consider effective pain relief strategies as the longevity of PWSCI is improved so that PWSCI are not forced to live longer with pain.

There is evidence to support that the experience of pain is worse than the consequences of SCI itself [40, 41]. Pain limits PWSCI far beyond the restrictions of the injury, not only physically, but emotionally, mentally, and socially, resulting in health dissatisfaction [7, 8, 42]. The experience of pain etches deep beyond physical affectations into the mental and emotional realms. Pain is an emotional experience and results in brain changes affecting the emotional response to external stimuli, influencing mood, and ultimately favouring the development of mood disorders [14, 42, 43]. Anger is a common emotion in people with chronic pain, although many individuals find it difficult to admit it due to anger being seen as socially undesirable [42]. Anger can be directed at themselves or others (such as family and the healthcare system), and the cause-effect relationship between anger and pain can be likened to the relationship between pain and depression [42]. Another cause-effect relationship our participants reported was the relationship between pain and muscle spasms. Callaway et al. [39] found that 69.7% of their participants reported pain related to spasms. Contextualised results regarding pain and spasms would be beneficial, as international studies support the strong relationship between pain and spasticity, as well as the perceived impact on life [44].

In our study some participants said that they pushed through the pain, similar to findings by Turner et al. [45]; however, their concentration was affected. Pain is an inherently attention-demanding sensory process and often results in affected individuals reporting attention deficits [46]. Individuals with chronic pain also show memory impairments, increased fatigue levels, slow reaction times, and compromised emotional decision-making [46]. The threat of severe pain often captures attention in a way that people who experience the pain find it difficult to disengage from it [42]. Different coping strategies should be taught to affected individuals so that they are better able to cope with their pain. Pain management techniques that focus on shifting the attention from pain, reducing catastrophizing, and improving resilience may be beneficial to ultimately reducing pain experienced. Pain is a well-known stressor, and unfortunately, when we are stressed, we are more likely to experience chronic pain, which creates even more stress, and more pain with no end in the cycle [42, 47, 48]. Various risk factors are reported including depression, chronic pain, and moments of crisis such as financial problems [49, 50]. Spinal cord injury predisposes individuals to established risk factors for suicidal ideation, even without the presence of pain [51, 52]. Experiencing a sense of isolation is strongly associated with suicidal behaviour, and, unsurprisingly, our participants expressed suicidal ideation. Suicide ideation is the thinking, considering, and planning of suicide and results from the combination of emotional factors such as feelings of isolation and alienation from others, low moods, and hopelessness [53]. In the able-bodied population, men under 50 years are more associated with severe suicidal ideation than men over 50 years [53]. Suicide is three times more common in PWSCI than in those without SCI [54], and there are over 700 000 people without SCI who commit suicide annually and many more who attempt suicide [50]. This then suggests that pain management needs to be an integrated effort to also include psychological assessment and management for PWSCI [55].

Our findings echo literature that emphasizes how pain is a hindrance to most tasks of daily living [7, 39]. People with SCI who experience high-intensity pain are more likely than others to experience frequent interference with activities of daily living, including sleep disturbance, and thus makes it difficult for PWSCI to achieve an acceptable QOL following their injury [37, 56, 57]. The quality of sleep of PWSCI with continuous pain is poorer than those without pain or intermittent pain [58]. More recently, Carlozzi et al. [59] found that better sleep quality was associated with better health-related QOL and that pain is worsened after a night of poor sleep. There is a bidirectional relationship between poor sleep and chronic pain as poor sleep may exacerbate pain and vice versa [60]. Similar to the literature, we also found that pain impacts social activities and functioning at home [38]. People with SCI in our study refrained from fully engaging in their communities or sporting activities due to the pain, in tandem with findings by Piatt et al. [61] and Fuseini et al. [1]. Pain interference has the power to negatively influence the QOL of PWSCI [62], who found that the QOL of those without pain was similar to their able-bodied counterparts.

Thinking about how pain might affect their future was a daunting task for some of the participants in this study, as they were unsure if they would ever attain pain relief, which is akin to the findings by Hearn et al. [63]. It is common for people with pain to be anxious and worried about their futures if the pain will increase, and the symptoms result in progressive disability, less independence, and the ability to work [42]. The worry about intimacy and possibilities of reproduction were reported in our study and the uncertainties of what the future holds for women with SCI living with pain are warranted, especially if the necessary education was not provided. The presence of chronic pain after SCI is not related to sexual function, and most women do not lose their ability to ovulate, menstruate, and have children after SCI [64]. Education on sexuality after SCI is an important aspect of rehabilitation, which is not always emphasized in the South African health system [65]. Family and social support after SCI is important and is associated with positive outcomes, such as reduced pain severity, increased participation in prescribed exercises, and higher satisfaction in physical functioning [1, 7, 66, 67]. However, we must note that it is not merely the presence of the support, but rather the quality of the support that will determine how PWSCI perceive their level of support [67]. Although family support is an important factor in SCI pain management, the focus is not on the family's management of a patient's pain but rather on the patient's self-efficacy to improve their health status [68]. It is therefore advantageous to involve PWSCI in pain self-management programmes as they would have improved perceived control over their pain, which is associated with better mental health [10].

Having hope in the future even amid pain suggests a level of resilience and prospects of the necessary levels of self-efficacy to manage pain. For example, some of the participants in this study did not see pain affecting them in the future as long as they took the pain medication. Hearn et al. [63] found that PWSCI who felt that pain was manageable felt able to make plans and was associated with taking a more active approach to pain management. Older PWSCI tend to be more accepting of living with pain, with Day and Thorn [69] suggesting that older people tend to catastrophize less and accept pain with advancing age. Pain catastrophizing has the potential to impact an individual's behaviour, functional ability, and mental health [10, 70]. This impact is because magnifying the pain exaggerates the threat of pain and ultimately increases pain by increasing the attention focus on the pain and the emotional reactivity to the pain [71]. Distraction therapy may therefore be a useful tool to enhance pain management [72].

## 5. Strengths and Limitations

The interviews in this study were conducted in the participants' homes, giving them the freedom to express themselves as freely as possible. Rich information was attained; however, the purposive sampling resulted in all the included participants having a traumatic SCI. Although traumatic causes of SCI far outweigh nontraumatic causes [73], there is a possibility that traumatic events may play a role in how we experience pain, and, therefore, people with nontraumatic SCI may report a different perspective on life with pain. Furthermore, there is the possibility that different experiences may have been reported by participants who resided more than 150 km from their discharging hospitals. During the data analysis process, two external academics with prior experience publishing qualitative studies were included in the coding process and not persons with SCI. Lastly, our study population was delimited to manual wheelchair users with paraplegia and we, therefore, caution against generalizing these results as more diverse responses could be possibly found in a more diverse population. Nevertheless, we believe the findings from this study may be useful in adding to the available literature on the experience of living with pain after SCI.

## 6. Conclusion

The findings in this South African study do not paint a different picture to global findings, suggesting that the experience of pain after SCI is similar globally. Pain is debilitating and worsens with more years of living with SCI. Pain lurks in the background and fluctuates in intensity while affecting physical, emotional, and mental health. Pain interferes with activities of daily living, social relationships, and productive work. Although living with pain brings uncertainty about the future, it also gives rise to a level of determination to try and live life to its fullest.

## Figures and Tables

**Table 1:** Sociodemographic and pain information.

Pseudonym	Gender	Age	Years living with SCI	Cause of injury	Employment type	NLI	Completeness of injury	Number of painful areas	Most painful location	Pain severity	Pain behaviour	Type of medication(s)
Aaron	Male	43	2	MVA (driver)	Unemployed (government disability grant)	T9	Complete	2	Lower back (below NLI)	8	Burning	NSAIDs
Abigail	Female	42	13	MVA (passenger)	Sales consultant	T6	Complete	2	Back (injury site)	6	Sharp	Opioids
Angela	Female	58	9	MVA (passenger)	Registered nurse (office work and counseling)	T10	Complete	2	Dominant shoulder	6	Sharp	Not on any pain medication
Abel	Male	39	6	MVA (driver)	Unemployed (government disability grant)	T6	Complete	2	Lower limbs (below NLI)	5	Pins & needles	Paracetamol & anticonvulsants
Abram	Male	37	6	MVA (passenger)	Unemployed (government disability grant)	T11	Complete	1	Lower limbs (below NLI)	10	Burning	Opioids, anticonvulsants, & tricyclic antidepressants
Adam	Male	60	4	Object fell from height	Unemployed (government disability grant and WCA)	T12	Complete	1	Back (injury site)	5	Cramping	Not on any pain medication
Adrian	Male	62	39	MVA (driver)	Administrator	T6	Incomplete	2	Buttocks	5	Pins & Needles	Not on any pain medication
Amanda	Female	42	12	MVA (driver)	Assistant director	T12	Incomplete	1	Lower limbs (below NLI)	8	Burning	Opioids, anticonvulsants, & tricyclic antidepressants
Albert	Male	50	4	MVA (driver)	IT specialist	T12	Incomplete	4	Lower back (below NLI)	6	Deep pressure	Opioids, anticonvulsants, & SNRI antidepressants
Alice	Female	32	17	PVA	Maintenance controller	T12	Complete	2	Upper back (above NLI)	8	Sharp	Opioids & tricyclic antidepressants
Alex	Male	45	8	MVA (passenger)	Unemployed (work disability grant)	T5	Complete	1	Lower limbs (below NLI)	10	Burning	Not on any pain medication
Andrew	Male	34	4	MBA	Unemployed (government disability grant)	T7	Complete	4	Lower limbs (below NLI)	7	Pins & needles	Not on any pain medication
Andries	Male	60	13	MVA (driver)	Pensioner (work pension)	T6	Complete	3	Back (injury site)	6	Sharp	Not on any pain medication
Anthony	Male	30	4	Gunshot	Entrepreneur	L1	Complete	1	Lower limbs (below NLI)	6	Electric shocks	Opioids
Archie	Male	23	5	Gunshot	Sales assistant & data capturer	L3	Incomplete	1	Lower limbs (below NLI)	4	Electric shocks	Paracetamol & NSAIDs

MBA = motorbike accident; MVA = motor vehicle accident; NLI = neurological level of injury; PVA = pedestrian-vehicle accident; NSAIDs = nonsteroidal anti-inflammatories; SNRI = serotonin-norepinephrine reuptake inhibitor; WCA = workers compensation assistance.

## Data Availability

Data for this study can be made available from the University of Pretoria's physiotherapy department at a reasonable request.

## Supplementary Materials

Supplementary Materials The interview schedule used in the study was developed by Hearn et al. [35], and modified by the authors to suit the South African context for this study. This paper reports findings on the questions “Tell me about your experience of pain since your spinal cord injury”, “What is your life like since experiencing SCI-specific pain?”, and “How do you think your pain will affect your future, if at all?”.
