# Supplementary material for: Exploring the Experience of Living with Pain after Spinal Cord Injury: A Qualitative Study
Source: Behav Neurol. 2024 Feb 2;2024:9081530. doi: 10.1155/2024/9081530 (PMC10857878; doi:10.1155/2024/9081530)
Supplement: Supplementary Materials — The interview schedule used in the study was developed by Hearn et al. [35], and modified by the authors to suit the South African context for this study. This paper reports findings on the questions “Tell me about your experience of pain since your spinal cord injury”, “What is your life like since experiencing SCI-specific pain?”, and “How do you think your pain will affect your future, if at all?”. [file 9081530.f1.docx]

# Interview schedule (modified from Hearn et al. 2015)

| **Open-ended questions** | **Probing questions** |
| --- | --- |
| 1. Tell me about your experience of pain since your spinal cord injury. | - Where is it located?  - How does it feel at best/at worst?  - How often does it present itself? |
| 2. How have you been informed about your pain? | - Was this helpful? |
| 3. What techniques do you use to cope with your pain, if any? | - What is the most effective strategy for managing your pain? |
| 4. What is your life like since experiencing SCI-specific pain? | - How does pain affect your everyday life?  - How have others reacted to your pain?  - Are there any activities you do differently now as a result of your pain? |
| 5. How do you think your pain will affect your future, if at all? |  |
| 6. Is there anything else you would like to add to the discussion? |  |
